# Yuri’s Story: Memory, Relational Healing, and the Reflexive Logics of Art Therapy in Japanese Clinical Psychology

**DOI:** 10.1007/s11013-025-09916-5

**Published:** 2025-06-02

**Authors:** Christopher Chapman

**Affiliations:** 1 Present Address: School of Social Sciences, Nanyang Technological University, Singapore, Singapore; 2 Institute of Social and Cultural Anthropology, University of Oxford, Oxford, UK

**Keywords:** Narrative, Care, Therapy, Subjectivity, Memory

## Abstract

Mental health care is a vibrant part of child protective services in Japan, and the adoption and utilization of psychotherapeutic techniques from abroad mark a complex site of cross-cultural exchanges. This paper explores how art therapy has been brought into Japan’s protection system and its implications for professional practice. Focusing on clinical psychologist Yuri and her narratives on learning art therapy, this paper utilizes an interpretive and phenomenological framework to illustrate the importance of embodied experience in delivering care and how practitioners may reform their perspectives on care by reframing their own traumatic memories. Yuri’s art therapy offers a culturally contextualized view of the self as social, care purpose, and resilience.

## Introduction

On a cool autumn afternoon, I entered the main office of the Juniper City Child Guidance Center, the local authority responsible for managing abuse investigations, mental health evaluations, and clients’ cases. The office was quiet save for the soft rustle of typing, the hum of air conditioners, and the chimes of ringing phones. Caseworkers were busy at their computers and stacks of paperwork, manila folders, and other records were piled high on their desks. Opaque windows gave the gray room a twilight ambiance. Yuri, a senior staff member and publicly licensed psychologist, came up to greet me. “How are you today?” she asked with a smile. “You’re right on time,” she continued, “I just finished a therapy session with a young girl. Let’s go to the therapy room and I’ll walk you through the techniques.” Later, Yuri explained, “This week we worked with children who experienced very difficult things—physical abuse, sexual assault, and one child’s parent recently died—the therapy we provided is important so that children can recover.” I asked after her well-being, “That sounds like it must be stressful, no?” Yuri replied, “Oh yes, it is. We need to trust in our training and tools. That’s why I try to keep up with new ideas in therapeutic practice. Have you heard of art therapy? It is a technique I have found to be very effective, although in a way you might not expect.” Clinical psychologists in social services, like Yuri, engage with real, messy, and sometimes unresolvable care issues. One matter at stake, then, is how others’ distress and suffering may bear on health practitioners’ embodied experiences and senses of well-being, prompting questions about how health practitioners take care of themselves.

This paper focuses on experiential healing and well-being as they emerge through a mental health practitioner’s training and reflection on a novel therapeutic tool, art therapy. I adopt a person-centered approach because of the self-focused sentiments that discursively emerged through my interactions with Yuri.^1^ Her performative narratives index the recollection and resolution of painful memories that, as I will discuss, blur a clear distinction between patient and healer, framing betterment as an interior but relational process. Yuri’s subsequent shift in professional worldview through art therapy, though, suggests how therapeutic innovation relies on cultivating healer empathy more than implementing novel care techniques with a patient. Overall, Yuri’s narratives on childhood memory, self-transformation, and carer purpose contrast a mainstream, neoliberal ethic of therapeutic practice, centering Yuri’s story as a keystone for better appreciating the social and cultural dimensions of medicine. This paper begins by reviewing anthropological scholarship on narrative and interiority, followed by a description of the research methods and background information on the overarching research. The paper then moves into a discussion of how Yuri’s story, as a set of narrative assemblages, conceptualizes the role of reflection in art-based therapeutic intervention and the carer/patient divide.

## Conceptualizing Interiority and Narrative

“It is interesting how our experiences stay with us—mental maps floating in our heads and nostalgic feelings tugging on our hearts,” Yuri explained. She was reflecting on the potency of memory in relation to the bodily senses, including how embodied experience, through memory, can channel sensory returns to one’s past. Yuri continued, “I remember being a young girl in a Seibu department store in the 80s, looking at the new movies with my mother, the aisles, and the people … all the pretty [city] lights passing me by in the back seat of the car as my father drove us home.” Yuri described how vivid memories can endure in the body, providing an affective and narrative window into self and society. “That store is long gone, but […] I can see it as clearly as I see you,” she stated. In Yuri’s professional life, corporeal experience and memory comprise primary matters of concern as she cares for abused and neglected children, a context that may invoke, in contrast to the quotes above, unpleasant but still equally vivid recollections and feelings. And yet, as I will discuss in this paper, the boundaries between patient and carer are not always clearly defined or stable. In a different conversation, Yuri mused on an open-ended question: was her professional trajectory a quest to reconcile some inner, embodied struggle leftover from when she was young? I pose this question without answer as a preface and provocative point to consider a speckled portrait of a person’s well-being and therapeutic potential: the complex relationship between the body, inner and social processes, time, aging, and memory, and the transcultural, formal or informal, contexts of (health) care practice (Chapman, 2025; Kirmayer & Gómez-Carrillo, 2019; Mills, 2014; Street, 2014).

Situated in broader anthropological scholarship on intersubjective well-being, this paper develops two elements, interiority and narrative, within the context of artful therapeutic practice to develop an understanding of the reflexive logics of mental health care vis-à-vis self, time, and affect (Biehl et al., 2007; Black, 2018; Kleinman, 1988; Mattingly, 2010; Rubinstein, 2016). Susan Hogan and Sarah Pink (2010, 158) theorize interiority, describing “inner dialogue, imaginative worlds and emotional reverie” as fluid processes rather than static states (see also Kavedžija, 2021). My focus on interiority centers memory as a primer for emergent discursive and affective fields within a context of psychotherapeutic engagement. Narrative is well established as a key site and way of making sense of one’s social world (Bruner, 1991; Garro & Mattingly, 2000; Jackson, 2008; Mattingly, 2010; Reck, 1983). These two concepts are useful to theorize here because Yuri’s art therapy reflections consisted of thoughtful narratives articulated alongside visual-material performances involving her handmade artworks, narratives that spoke of Yuri’s social world through inner monologues indexing her present work life and her remembered childhood. Drawing on this context, I propose an introspective frame of (self) relational healing that contributes to contextualizing what and how resources are mobilized to manage personal well-being, a process that Catherine Panter-Brick (2014, 432) frames as resilience.

In this paper, I draw from Cheryl Mattingly’s (2010, 41–45) frame of narrative phenomenology as it links social action to sense and sensation, posing narrative as dramaturgical more so than textual. Narrative is an important analytical base because art therapy incorporates the whole body and its sensory potential into an interactive story of self and other. Uniting narrative with interiority, then, helps to bring inner dialogues, imaginative spaces, and affective changes into an interactive present that may further mediate subjectivity. Intertwined with this idea of narrative interiority is Mattingly’s (2010) suggestion of hope as a paradoxical process of potential and limitation in the context of stakeholders seeking betterment. The hope of betterment, she offers, promotes creativity and imagination in stakeholders, from family members to health professionals, as they navigate a complicated landscape of technologies and services that index a multitude of different, unknown futures. When distress, illness, or tragedy impact one’s life, narrative offers a tactical way to reorder events and reframe the meanings of unwanted situations (Chapman, 2020; Kleinman, 1988; Ezzy, 2000; Das & Das, 2007; Good, 2000; Frank, 1995). Narratives on health and illness as multiple have also pointed to important distinctions between patient and carer; practitioner well-being offers a productive avenue for exploration.

Plot is a key characteristic of narrative as people (patients) may reorder events surrounding their illness or distress into a cohesive story that has a logical rationale of cause and effect that may be distinct from others’ discursive understandings. I specifically situate the clinical context of narrative as a focus of this paper, finding insight in Mary-Jo DelVecchio-Good’s (2007, 367) clinical narrative as a tool to highlight the interactive dynamics of patients and clinical specialists through long and complicated care journeys (see also DelVecchio-Good & Good, 2000). However, narrative should not be taken as an index that resolution and well-being are stable, certain, or reliable processes. Paul Ricoeur (1990, 207) suggests that narrative may be “an open-ended, incomplete, imperfect mediation… the network of interweaving perspectives of the expectation of the future, the reception of the past, and the experience of the present.” Embodied experience and story intertwine, and subjectivity becomes a performative project. Narrative may become less about a cohesive plot or canon, then, and more about possibility and an orientation toward the future and betterment that indexes the multiplicity of interests that shape subjectivity—how one is subject to social and political forces (Jackson, 2008, 31). Narratives in this complicated frame are enmeshed in their context(s) of enactment; they impart shifting perspectives, pressures, and intentions—a kaleidoscopic look into how people navigate a social world (Carrithers, 1995). And as a performative process, betterment and resolution take time, work, and engagement. Yuri’s inner journey to remembering and resolving inner pain comprises a set of non-linear and irregular, intervallic narratives.

## Methods and Ethnographic Background

This paper draws upon eighteen months of ethnographic research on caregiving and subjectivity in Japan’s child protection system.^2^ As this system is decentralized, I conducted multi-sited fieldwork at child protective services offices, group homes, and foster homes within Juniper City, a special ward of the Tokyo metropolis. This paper focuses on my engagements with child protective services at the Juniper City Child Guidance Center where I shadowed caseworkers and psychologists. The Juniper Child Guidance Center was built in the early 1990s and serves a population of 900,000 in the city and surrounding towns, supported by a variety of sub-offices. There are approximately ninety full-time employees.

I center my discussion and analysis on Yuri, a clinical psychologist in her early forties with over 20 years of experience.^3^ She grew up with her mother and father in northern Japan and moved closer to Tokyo to begin an undergraduate degree in psychology. After completing her master’s degree in clinical psychology, she accepted a job offer from the Juniper City Child Guidance Center. Currently, Yuri is the supervisor of a psychology team that supports the case management section, though she occasionally assists the investigation team by conducting interviews or providing her input. Our interactions consisted of several formal interviews, an extensive life history interview, and informal conversations at the office. She is not as vocally critical of the child welfare system as the other staff but was interested in improving her knowledge and skills to better support children in need. The main job of a child guidance center psychologist is to conduct tests and assessments on children. Many of the other mental health professionals I met upheld these tasks as their primary duty. Yuri, however, was more interested in therapeutic support. To aid in my understanding of how mental health practitioners care for children, Yuri taught me the basics of art therapy, which has become the cornerstone for how she envisions progress in child welfare. She is proud of her profession and hopes that new developments in treatment will help her to take better care of children in need.

The history of mental health support in child protection provides an important background context for Yuri’s story. The formal implementation of mental health care within the child protective system in Japan is recent, having been formally established in 2000 after a drastic rise in reports of child abuse and neglect (Chapman, 2024a, 70–78). The national government recognized that children’s health and well-being were at risk of socio-developmental harm and mandated the placement of mental health providers in child protective services to assist with casework and therapeutic support. The broader research this paper is based on explores the effects of this change on the social, spatial, and affective dynamics of caregiving (Puig de la Bellacasa, 2017; Cook and Trundle, 2020; Danely, 2014; Sweis, 2021). In Japan, clinical psychology as a profession has undergone several changes throughout the past twenty years, notably the enactment of varied pathways toward professional certification and national licensure. The adoption of psychotherapeutic techniques has also shifted in line with practices abroad, with tools like cognitive behavioral therapy having been translated from the United States. These changes speak to domestic scholarship and clinical practice which have taken up and locally reshaped psychological thought throughout the past century (Kitanaka, 2003; Ohnuki-Tierney, 1984; Ozawa-de Silva, 2006). Art therapy, though not new or standardized as a therapeutic option within child protection, represents a niche tool deserving of closer examination because it is a narrative nexus of health innovation, subjectivity, and cultural nuances of care.

Art therapy is a psychotherapy approach used to explore, communicate, and reshape one’s emotional state using artistic and creative tools such as drawing, photography, writing, painting, and more. While some care practitioners were concerned with results, others I spoke with suggested that art therapy is more about a creative process of artmaking which, in turn, may have therapeutic benefits irrespective of time or place. Art therapy has roots in 20^th^-century England, arising out of mental health care institutions where patients were able to express themselves in a way that broke down the barriers of institutional confinement.^4^ Art, such as painting and drawing, became a vehicle for creativity and emotional release. Professional organizations focused on art therapy education and practice emerged in the U.K. and U.S. in the 1960s with others following in Australia, Singapore, and New Zealand. Art therapy currently holds an established position within mental health care and is one of many therapeutic tools that care practitioners may utilize in clinical spaces around the world (see also Pells et al., 2022).

In Japan, art therapy has a strong social history starting in the postwar era that indexes the therapy’s local malleability as people integrated regional practices such as painting, calligraphy, flower arrangement, and more into therapeutic pathways (Mizushima, 1971; Ono, 2017). Literature from abroad, like Cathy Malchiodi’s *Breaking the Silence: Art Therapy with Children from Violent Homes*, was also translated into Japanese and circulated among educators and practitioners (Narahara, 2010).^5^ Within Japanese child protection, art therapies hold a notable position as sand-play therapy is often visible in child guidance centers and child protection institutions. Sand-play therapy represents a link to the integration of mental health support in child protection in 2000 as, alongside the employment of practitioners, this therapy was mandated as a tool for official use (Enns & Kasai, 2003). Art therapies outside of play therapy, however, are sporadic in their utilization and not standardized. Yuri suggested that artistic and creative therapies are hardly utilized at all within child protective services. The sand-play table in the Juniper Child Guidance Center, for example, sat covered and untouched throughout the entirety of my fieldwork, and Yuri stated that the staff had not used it in years. Nevertheless, Yuri was keen on learning about new practices and ideas to innovate her professional toolkit, which is the main reason she agreed to participate in my project: to learn about how ethnographic approaches handle care, health, and personhood.^6^

## Remaking the Self Through (Art) Therapy

In this section, I explore how Yuri taught me about art therapy in terms of a pedagogical and performative narrative that indexed embodied knowledge as a quality of being a carer. Sitting down in the therapy room at the Juniper Child Guidance Center, Yuri explained how she learned about art therapy, the effect it had on her, and how she teaches it to others:

“Art therapy is like serving a meal. You [the therapist] can’t serve something that you have never tasted before and say [to your patient], “It's delicious.” So, the therapist needs to experience different materials and get to know them well. Art therapy means taking care of a person by carefully observing their creative process. This must be done within what I call the ‘therapeutic container.’ Otherwise, it ceases to be therapy. By ‘container’ I mean the protective and enveloping action of the therapy. Both the therapist and the client enter this container, but it’s the therapist’s responsibility to make a comfortable space for the client. Something which I think a lot of psychologists in child protection forget is that therapy is, essentially, about *caring* rather than *fixing*. Care has to do with noticing a person as a person and tending to or looking after them in a way that strengthens their spirit. This is how I feel about my entire art therapy experience to date.”

Yuri’s narrative speaks by way of contrast to the professional disposition of mental health care in Japan’s child protection system, suggesting a window into technical innovation, subjectivity, and healing. Yuri’s art therapy highlights a strong narrative quality and incorporation of material objects into therapeutic work, suggesting that direct experience—of doing the therapy—marks a key affective qualification of being an art therapist. This instance of reflection aligns with Mattingly’s (2010) dramaturgical framing as Yuri’s narratives on therapy and sense of self were spoken alongside a visual-material demonstration of her artistic creations, and this ethnographic exchange was mediated through performative and “generative” interaction (McGranahan, 2020).

Yuri’s narrative expresses the importance of embodied knowledge, of *doing* the therapy as a way to know it and being able to use it to care for others. Becoming a therapist, from her point of view, is a process of experiential learning. Her explanation of the therapeutic container as a safe expressive space indexes the aforementioned concept of interiority (Hogan & Pink, 2010, 158). For the client, therapeutic action rests on the sensory context that the therapist helps shape by subtle ways of interacting, moving, speaking, and listening. The focus on “caring” rather than “fixing” eschews a rigid, medicalized lens, yet Yuri said that she still tries to see her clients’ actions within the language of her profession—discourses on child abuse, psychosocial development, and intervention. What Yuri does not do, however, is take copious notes on how children and young people behave. Instead, she sees art therapy simply as therapeutic time solely for the client. Notetaking, she said, interrupts the “therapeutic container” because the client can “obviously see what the therapist is doing.” What matters is the affective interaction between client and healer through the artwork as an intermediary object, as Hogan and Pink (2010, 158-9) explain that crafting an artwork and ruminating on it can lead a participant into inner dialogues, and the tactile, material qualities of the crafting process may also invoke moods, desires, and instinctive embodied emotions. These states of being are fluid more so than static or fixed, and the facilitator aims to bring these interior feelings to the “surface.” In the context of child protection, Yuri understands that she and the children may navigate complex and sensitive topics, ranging from abuse and disability to questions of missing parents and confusion about social services. While I list these topics here plainly, it is important to note that they can be emotional, messy, and sometimes difficult to reconcile. Knuckle cracking bone, fearing a father’s touch, searching for dinner in an empty refrigerator, hearing a mother tell you that it would have been better if you were never born. These are some of the stories that came up in practitioners’ day-to-day work and engagements with children. In art therapy, these are the kinds of interiorities that Yuri seeks to bring to the surface so that they be made workable and, thus, resolvable.

The first step in learning how to be an art therapist, Yuri explained, is by creating something on your own and enmeshing it within a story:

“The process of creating my own story was very important to me. It was just the [teacher] therapist and me, and through forming my object, of thinking about the emotion I was trying to bring forth, I felt secure in a protected space and time—a place that could truly be called a ‘therapeutic container.’ Whatever the material was, whether tissue art, pastel (play with colors), pocket dolls, spheres, ink, guided meditation, mobiles, etc., I would try to explore the image I had in my mind and find the feeling that fit it perfectly. After creating something, I would share it with the therapist, which became a way for me to talk about what was going on within me. The object became ‘me.’ Some things only come into view when we see ourselves from the outside. It was very important to me that I was the [narrative] lead because it was my work. The art therapist would watch over me and give me hints when I was lost or shine a little light around me. They mostly just listened to the various things that came to my mind. Rather than saying something, it's more like they were there for me so that I can face the matter on my own, with support if I need it.”

Yuri speaks of interiority, and yet the creation of an artwork is a powerful social moment because of the cooperative work done between the client and the mediator. Sharing one’s inner world through meaningful objects brings affect and spoken narrative into being through performative action, and an engaged person watching, listening, and helping someone care for themselves situates art therapy as a collaborative endeavor. It is this social experience, Yuri suggests, that articulates how becoming an art therapist is best learned by experience. This orientation to therapeutics frames healing as a process that seeks to resolve distress and suffering. The teacher’s narrative in this interaction is a clinical narrative that, as DelVecchio-Good (2007, 369) writes, “weds the experimental to the therapeutic … [inscribing] treatment experiences on a patient’s psyche and soma, under the guise of multiple plots and subplots that the professional subspecialties envision for patients and clinicians.” The use of oneself in the patient role as the explorative and incisive ground of pedagogy, though, marks a strong contrast to non-psychological therapeutic training. Moreover, there may be, as will be discussed, unexpected consequences.

Providing effective or empathetic care may emerge as a skill achieved through bodily action. The therapist’s attention and body are oriented toward the interactive body-artwork assemblage, centering on the client’s sense of self as an object of inquiry. Yuri was surprised with how introspective the process became since it was participant-led. At her teacher’s light suggestion, she reflected on her self-identity through a particular emotion and grappled with it while making a creative artwork.^7^ Yuri understood through her bodily senses what a “therapeutic container” was and how it should feel. Learning art therapy by doing became a process of making oneself vulnerable to another by making one’s inner dialogues and emotional states known to or perceivable to others. This way of feeling and sensing is the embodied understanding that her teacher wanted her to acquire and refine so that she could facilitate the process for others. In a way, then, the therapeutic training exercise, as a personal yet socially revealing interaction, shapes an imagined social future—a future where one will be facilitating a therapeutic experience for someone else, an unknown other. Yuri’s training hints at an alignment with Hogan and Pink’s (2010, 166) outline of a feminist, social art therapy which posits that self-transformation does not occur in isolation, but is enmeshed within social structures, cultural discourses, power imbalances, and historically shaped institutions. Yuri’s art therapy, consequently, provides a speckled view into subjectivity—the individual as subject to broader social contexts—and the position of therapeutics, a topic I explore next.

## Being a Better Carer Through Self-Transformation and Empathy

Learning about art therapy with Yuri shifted from a solely pedagogical exercise into a conversation about her life history and, notably, a story about her worldview as a health practitioner in relation to painful memories. In this section, I explore the position of the facilitator in art therapy, focusing on how the potential of being a therapist may rest on the reframing and resolution of pain and distress, adding a layer of complexity to the analytical point that being an art therapist relies on embodied knowledge and opening one’s inner world to others’ perception. Yuri’s personal, paradigmatic shift began when she recounted a notable artwork she created:

“Of these [the mediums and tools used in art therapy], the one that was particularly important to me is difficult to pick out, but I would have to say that a mobile I made was the most important. I showed you it before, but it is a mobile with five stones suspended and balanced on a string. When I made this, I made it while thinking about ‘facing my wounds,’ which is a theme my teacher gave me. The place was usually, but not always, my teacher's studio, a small house with a fireplace. During our training sessions, there were two teachers and two or three students. I knew them well, so I felt quite comfortable with the place and the group. So, I was able to ‘face my wounds.’ The idea of the exercise was to make mobiles, but you could use whatever you wanted to make them. I was thinking I would like to use a stone of some weight, say, 2–3 cm in diameter. Just then, the teacher brought some stones from the back of the studio, and I looked for one that looked good. This center stone [displaying the mobile] had a rustic ‘me-ness’ feel. It was not particularly beautiful, but smooth and well-rounded. I chose it. I wrapped it in a little bright yellow braid and decorated it. The four surrounding stones are: a white beautiful but slightly angular stone, a black rounded stone, a beige-ish rustic stone, and a greyish stone. All these represent ‘me-ness.’”

These colored stones, Yuri explained, represented different self-images—a collage of the diverse ways she imagined herself to be. Each one spoke to a different contour of her embodied experiences from childhood to the present, from failures to successes and bright memories to forgotten encounters. Yuri affirmed the center stone as one reflection of how she saw herself: “not particularly beautiful, but smooth and well-rounded.” The theme of her training, though, was ‘facing one’s wounds.’ Wound in this context refers to interiority—an intimate memory of a painful or distressing experience. Yuri explained:

“I chose the greyish stone as ‘my wound.’ The wound I chose was a very memorable time. It was when I was a young girl in junior high, when I was harassed and bullied at school by other kids … it went on for a year and I hid it from my family. I was initially thinking of choosing a dark brownish thread to hang the stone from, but the stone said to me, ‘I (the stone/my wound) am a part of you, so take good care of me!’ So, I changed it to a colorful thread. The threads were beautiful, but they were bushy, tangled and they felt thin and insecure. I felt thin and insecure. I started to think of my ‘wound’ in such a way that it became known. I felt that my ‘wound’ was an important part of me. I still wish I had not had the experience that led to that wound, but I cannot separate it from me. So, to be able to think of that pain as an important part of me was a tremendous change. Instead of being a painful memory that I tried to forget about, I instead think of it as a companion. I keep [the mobile] at home. My wound is not the sum of who I am—but a part of me just like the other stones. Dealing with it gave me control and freedom.”

A key theme emerges from this conversation with Yuri: how learning art therapy unearthed a painful memory and helped resolve it, a point that aligns with Taussig’s (1980, 7) definition of therapeutic resolution as a relational process distinct from medical nosology. This pain could potentially be framed as trauma, but in either case, it is the memory in the body that shapes affect (Kirmayer, 2015). Art therapy *can* be therapeutic, but Yuri’s position as a care provider adds a complicated twist to the role, motivation, and intention of care intervention. Yuri’s art therapy became a reflexive tool and care resource, complicating a clear picture of therapeutic intention and suggesting a form of (self) relational healing as Yuri’s training session reframed poignant childhood memories and her professional worldview.

Yuri’s introspective reconciliation as described above was not only a therapeutic moment, but a reorientation of her lifeworld as a therapist. I asked if she understood before her training that she might have to think about stressful or sensitive topics, and she said no. Both the painful memory and its resolution were unintended and unexpected. I found this to be a striking development in Yuri’s narrative, that her training unintentionally became a means of maintaining one’s well-being, that is, a resilience resource (Panter-Brick, 2014, 32). But Yuri challenged a stereotypical image of a cathartic, narrative arc with a linear plot. She was not searching for something beforehand, she felt, and she did not realize the means to resolve her trouble—it happened in the moment of feeling and articulation during the therapy training session. In our conversation, she also second-guessed herself. She was unsure. Why was she trying to help children in need? Her experience of being bullied and abused was a vivid memory for over thirty years. While Yuri admitted that she forgot about it because it did not come up in day-to-day life, she said that in situations where she did recall it, the memory was still potent. The art therapy brought this memory into a performative and discursive field that recast Yuri-as-child under the guise of a patient because she perceived her childhood self alongside her clients. As mollifying as her therapeutic resolution was, it also uprooted her professional sense of self as a care provider, further illustrating the self as socially malleable. She explained:

“There is a scene in a show called *March Comes in Like a Lion*. The main character, a high school student, helps a junior high school girl, whom he is friends with, when she is being bullied. He reaches out his hand to her. I had an image of myself, who was bullied in school, also being reached out to, and saved. With this therapy, in this sense, I want to try lots of different things to help troubled children, but at the same time, it also feels like I am trying to save the past me. But this just means that I am not tending to my own problems and don’t even realize it. When you try to tend to someone else’s pain, you may end up doing it to help yourself heal, and you may wonder, are you really doing it for the other person? The difference is paper-thin.”

Yuri’s position as a practitioner shifted, and she found new potential in helping others rethink their pain because of the transformative potential of her therapeutic experience. If embodied experience is a prerequisite for facilitating effective art therapy, then Yuri’s story also suggests that knowing pain, distress, and discomfort—along with betterment—may shape one’s orientation toward and ability to deliver care. As she mentioned earlier, care is not fixing, but subtle, empathetic ways of interacting and seeing a person holistically. Yuri supported the idea that the lived experience of distress can help shape empathy. Regardless of the motivation, I find it compelling how art therapy may be a mutually transformative process, centered here on Yuri’s fractal position as a care provider, patient, curious learner, and former child, among other fluid identities. By socially opening one’s emotional states—one’s interiority—as Yuri had, a person may realize the potential to explore, negotiate, and reconstitute conflicting senses of self (Hogan & Pink, 2010, 166).

## Therapeutic Reorientation and the Social Self: Reframing Patient and Healer

Yuri’s subjectivity speaks to broader challenges in Japanese society and the social care system. As she discovered, her therapeutic reorientation marginalized the healer’s position—who cares for the carer? I focus here on how Yuri’s story complicates the logics of a patient-carer relationship by constructing both roles as an interior-oriented narrative. Yuri’s self-discovery marked, in a way, a successful, if delayed, case of child protection in that she reconciled a pain stemming from her childhood self. Puzzling questions of the self, as Yuri explained, help set self-reflexivity and intersubjectivity as an important facet of delivering care:

“Connecting with oneself also means not ignoring one's own feelings. A child I worked with said, ‘I am the one who can really understand how I feel, but if I don't acknowledge my feelings, then I really am alone.’ Isolation, though, is not the same as loneliness, although ultimately, we are all lonely. To take on that loneliness, we need a part of ourselves that we can share with others, and the therapist helps the client take on that loneliness… It is important to create a ‘safe’ feeling in the moment of sharing so that when the client feels ‘a little different,’ they can tell me about it without reservation. I remind myself of this often, because I can easily get tense if I don’t feel comfortable.”

Yuri’s turn to loneliness invokes the broader context of contemporary Japanese society, which Ozawa-de Silva (2021) aptly describes as a “lonely society.” Japan’s economic downturn in the 1990s severely impacted people’s ability to realize a normative middle-class lifestyle and socially valued—and expected—life trajectories (see also Allison, 2013 and Kawano et al., 2014). This imagined lifestyle includes a university education, full-time work that can support a family, and a nuclear family living in a detached house in a suburban neighborhood (Chapman, 2024b; Nakamura, 2013; White, 2002). Currently, however, individuals increasingly live alone, work multiple part-time or contract jobs, and do not make enough money to support the normative lifestyle. Loneliness is a keystone of framing post-economic bubble Japan because it is difficult for people to create and sustain meaningful social relationships. This social reality reflects ongoing issues ranging from high suicide rates and low birth rates to an aging population and an entrenched conservative political system. Mental health services remain stigmatized and underutilized in Japan; psychotherapy, for example, is relatively uncommon and not fully covered by national insurance. Strict values around education, work, and family continue to pervade popular culture. In the context of child protection, the space Yuri works in, many of these social issues underlie children’s journeys into and experiences with the welfare system. Child abuse rates, as previously mentioned, have risen since the 1990s, illness and disability rates among children in care are on the rise as well, and work and education outcomes of children with care experience remain poorer than the national average (Chapman, 2025, 19–20). Yuri’s subjectivity references this darker side of society; it is little moments of exclusion, like being bullied at school as a child to little moments of healing, like reconciling a thirty-year-old painful memory, that speak to broader structural formations and their contradictions that care clients and practitioners must navigate.

The unintended but potent prospect of self-transformation, notably, is what Yuri finds enticing about art therapy. Hogan and Pink (2010, 166–170) state:

“…in art therapy we cannot undo discriminatory practices… but we can actively interrogate them … We are all subject to contradictory discourses and the wrenching between these is something that can be explored in art therapy … art therapy invites us to participate in ways of knowing in practice, acknowledging that such knowing will only be found in practice, but that even so it has the power to impact on things that are outside that actual moment of knowing and of practice.”

Yuri’s “wrenching” between not knowing if she is motivated to care for others or if she is seeking to care for herself speaks to loneliness in the sense of a lack or precarious presence of supportive social networks and well-being programs in her own life. Yuri further reflected on her art therapy training by musing that “it is interesting, and a little unsettling, to think that all I have done with my career might have been a long road to heal old wounds.”

Art therapy can be a means of mitigating the rigid institutions, social fragmentation, and exclusionary processes that a person in post-bubble Japan may experience. While this subjectivity is not distinct to child protection, it is pronounced because people involved with child protective services tread a social zone that subverts normative beliefs about kinship and belonging (Chapman, 2025). It is also pronounced because practitioners comprise the core of direct care services.

The unsettling of Yuri’s worldview, however, was not a cause for inner crisis, but a catalyst of renewed vigor. Yuri is enthusiastic about art therapy as an innovative tool for mental health care. Yet, her professional worldview is not driven by a need to share art therapy with other practitioners. Instead, Yuri finds that improvement is in the potential lifeworld shift itself—this is something she wants others to achieve, whether the tool is talk, art, play, or something else. Similarly, Yuri’s stance does not include a desire to provide art therapy to more children. Rather, her stance is that learning about art therapy and realizing self-reformation through it has fundamentally altered how she views “care.” Sara Ahmed describes that “the objects that we direct our attention toward reveal the direction we have taken in life […] orientations are about the directions we take that put some things and not others in our reach” (Ahmed, 2006, 546–552). Histories shape bodies, she suggests, and bodies invoke histories through (inter)action. While specialized skills are important for a clinical psychologist and psychiatrist, Yuri found it is more important that a person can listen to and be sensitive with a client. Innovation in caregiving, she said, is step-by-step—person-by-person—centered on the self. Yuri tries to be supportive of the children she works with so that they might have a similar shift in perspective, but, she finds that art therapy is a means for her to become a better carer by developing her capacity for empathy. Innovation in mental health care, consequently, is not embedded in new tools or techniques themselves, but in the transformative potential to reshape a care provider’s inner understanding of their subjectivity, to reorient and reinvigorate one’s sense of self and purpose by indexing painful experiences and resolving them. Yuri did not articulate her idea of therapeutic purpose along soteriological lines, but that every child and former child may have a thorny memory that could be unearthed and reframed as part of living a more fulfilling personal and professional life. But, this process can take time, sometimes long after children grow up. Her understanding of care, then, draws from and encapsulates both a desire to help children and help herself, blending registers of labor, affect, and ethics (Puig de la Bellacasa, 2017). These feelings do not need to be mutually exclusive or contradictory categories, but an intersubjective representation of the multifaceted, social-cultural self as expressed in the moment of making an artwork. Yuri shared:

“We don’t have the same scars, but we all have them. If you live your life, you will have new wounds every day. It is important to take care of yourself. If you ignore your own wounds, telling the other person to take care of themselves is not convincing at all. Caregivers and other professionals can help with [care]… it does not have to be professionals like me.”

Yuri’s vision of innovation is localized in a frame of interiority, but in analyzing this subjectivity, I find that art therapy also provides a space to ruminate on the practice of mental health care. Yuri lamented that care professionals, particularly other mental health practitioners, are not keen on utilizing psychotherapeutic techniques. She, for example, is the only psychologist at the Juniper Child Guidance Center who uses art therapy. I similarly observed that novel therapeutic approaches, like cognitive behavioral therapy, did not see widespread adoption (Chapman, 2024a, 169–174). Yuri said that this disinterest is not a matter of individual preference but of the profession’s orientation and its integration within the social care system. The system is comprised of rigid bureaucratic systems and driven by procedural precedent, and promoting innovation is often top-down due to the interest of upper administrative officials. The drawback, however, is that when those personnel rotate out of their posts every few years due to longstanding policies on civil service roles, their successors often do not continue implementing experimental or novel ideas. As mentioned earlier, clinical work, while on paper, does not constitute a child guidance center psychologist’s primary duty. Rather, administering developmental and psychiatric evaluations comprise the bulk of their work. Yuri’s introspection through art therapy, thus, also destabilizes mainstream practices of psychological care. While marginalized, her use of the therapy continues to unsettle what and how children—and care providers—engage with psychological medicine by providing embodied tactics to navigate and contend with inequities in the wider social world.

The element of relational healing in Yuri’s art therapy portrays well-being as not a matter of carer and patient, but a circuit upon which self and other coalesce into a social self, blending or blurring the boundaries between these fixed roles and states. Well-being, then, is best framed as an ongoing process of (inter)action, perception, and understanding in relation to others. Art therapy emerges as a potential route to accessing one’s inner world, supposing that therapeutic reconciliation is not embedded in the therapy framework itself, but, again drawing on Hogan and Pink’s (2010, 171) suggestion, in the intersubjective dialogue and coordination in the social moment. Situating well-being in frames of care, conviviality, and creativity, Iza Kavedžija (2021, 6) explains that by “looking at wellbeing through these three theoretical frames we become aware of the … entanglement with others in relations of care and dependence on others; and the temporal unfolding of well-being when looking at creative processes.” Art therapy training and practice provide a creative and convivial space under the banner of care and, within this well-being-mediating space, I posit that *therapeutic* and *therapist* potential is relational and affixed to the reframing of distress and suffering, redefining a binary of patient and healer (see also Kavedžija, 2019, 140–1).

Yuri’s story as a clinical narrative configures a patient-carer dynamic that envelopes the self within a sensorial, remembered world where new truths of one’s identity and subjectivity may be revealed through creatively artistic but intentional technical actions (DelVecchio-Good, 2007, 368). Yuri’s art therapy and its intersubjective elements comprise a nuanced set of resilience resources that may be channeled to sustain one’s well-being. These elements include the self, narrative as dramaturgical storytelling, creative materials, and the surrounding structural context of society and social services. The potency of art therapy, as discussed, is internalized, moving possibly from a clinical narrative to an embodied clinical monologue that Yuri’s clinical work invokes. This is notable because Yuri’s clinical duties are enmeshed in a wage-labor system, tasks that necessitate direct engagement with clients’ messy lives and distressing experiences, a context that marginalizes practitioners’ autonomy, health, and well-being. This structured position presupposes that the carer, intentionally or not, may risk examining their own storied life from an analytical perspective, exemplifying a dappled portrait of how others’ suffering is social and transcultural, as is one’s well-being (Wilkinson & Kleinman, 2016). The myriad social and affective forces that orient people into roles of patient and healer also confound these roles as the boundaries between the two may blur or blend when healers must contend with their own distressing experiences in the context of their care labor. The therapeutic potential invested through this style of interior-oriented narrative creates and brackets aspects of the self as a relational anchor from which to remember and rework meaning and distress. It is precisely this interior-social state of being both patient and carer that came to the “surface” in Yuri's artistic-clinical narratives, a cathartic process that in turn redefined her self-identity as a care practitioner and renewed her self-purpose irrespective of her professional training. Yuri’s story, then, invites an opening up of the boundaries, roles, and social dynamics involved within the dramaturgy of the clinical narrative and how care professionals manage their well-being in sometimes uninvited and emergent ways.

## Conclusion

This paper explored Yuri’s story of art therapy as an intersubjective encounter and relational healing process. Her recollection of a painful experience and the potency of an ability to reframe that memory, despite an unsettling of her professional worldview, demonstrate the happenstance potential of art therapy as a situated, reflexive, and mediative process. Healer Yuri’s orientation toward wanting to help children with care needs and the broader situation of being involved in emotionally heavy and stressful labor shaped an inclination for wanting one’s therapeutic work to be effective. Reflecting on her training in art therapy, Yuri realized a dual desire to help children and herself, a point that centers one’s inner world as the site of therapeutic discourse and perception. Interiority is a complex assemblage and the affective matter that comes to the ‘surface’ may be equally fractal, but also poignant reflections of embodied social life and cultural productions that mediate meanings of self and purpose. Yuri and her art spoke to a broader socially and historically situated subjectivity as loneliness marks a common experience in post-bubble, neoliberal Japan. In Yuri’s case, her art invoked questions of intention and feelings of uncertainty with respect to whom she was trying to help. The introspective nuances of Yuri’s training experience illustrated how a healer can learn to see oneself alongside or in place of a client by way of invoking vivid memories of one’s childhood self. Analyzing this point, I posited art therapy as a reflexive practice in that it reshaped the logics of therapeutic engagement by distorting the boundaries of the patient-healer dynamic. Art therapy and the potential for improving well-being was an intersubjective process, whereby a person reformed their social role and clinical position by becoming both patient and healer. This transformation was not a permanent shift but an emergent and embodied social form subject to further mediation. Overall, artistic therapies provide a conceptually fruitful space to consider the relationship between self, well-being, and medicine.

## Footnotes

1 This paper draws on Biehl’s (2007) person-centered approach to Catarina in Vita, an asylum for unwanted family members, focusing on how Catarina made sense of their social world, health, and displacement through fragmented, non-linear narratives.

2 For more in-depth information on child protection in Japan, please see Chapman, 2024a, Goldfarb, 2025, and King, 2020.

3 Yuri is a pseudonym, as are all people and place names in this paper. Yuri chose this name herself.

4 Art therapy has a long, storied history that exceeds the scope of this paper. If you are interested in this topic, please see Hogan, 2001.

5 Translated in 2002 into a book titled *Higyakutai-ji no ātoserapī: E kara kikoeru kodomo no messēji (Art therapy for Abused Children: Children’s Messages Heard through Pictures*).

6 Yuri was particularly interested in incorporating insights from ethnographic research with children into her clinical duties. For more information, please see Chapman, 2025.

7 Yuri’s explanation shares similarities with *naikan*, a therapeutic practice situated in Japan that centers on self-reflection as therapeutically meaningful. For details, please see Ozawa-de Silva, 2006.
